# Development of Metal-Ceramic Coaxial Cable Fabry-Pérot Interferometric Sensors for High Temperature Monitoring

**DOI:** 10.3390/s151024914

**Published:** 2015-09-25

**Authors:** Adam Trontz, Baokai Cheng, Shixuan Zeng, Hai Xiao, Junhang Dong

**Affiliations:** 1 Chemical Engineering Department, University of Cincinnati, Cincinnati, OH 45221, USA; E-Mails: trontzam@mail.uc.edu (A.T.); zengsn@mail.uc.edu (S.Z.); 2 Electrical and Computer Engineering Department, Clemson University, Clemson, SC 29634, USA; E-Mails: baokaic@g.clemson.edu (B.C.); haix@clemson.edu (H.X.)

**Keywords:** metal-ceramic, coaxial cable, Fabry-Pérot interferometer, sensor, high temperature

## Abstract

Metal-ceramic coaxial cable Fabry-Pérot interferometric (MCCC-FPI) sensors have been developed using a stainless steel tube and a stainless steel wire as the outer and inner conductors, respectively; a tubular α-alumina insulator; and a pair of air gaps created in the insulator along the cable to serve as weak reflectors for the transmitting microwave (MW) signal. The MCCC-FPI sensors have been demonstrated for high temperature measurements using MW signals in a frequency range of 2–8 GHz. The temperature measurement is achieved by monitoring the frequency shift (Δƒ) of the MW interferogram reflected from the pair of weak reflectors. The MW sensor exhibited excellent linear dependence of Δƒ on temperature; small measurement deviations (±2.7%); and fast response in a tested range of 200–500 °C. The MCCC has the potential for further developing multipoint FPI sensors in a single-cable to achieve *in situ* and continuous measurement of spatially distributed temperature in harsh environments.

## 1. Introduction

Because of the abundant reserve of low-cost coal in the USA and worldwide, coal-firing electricity generation will continue to be a major part of the power supply for a very long time. However, the adverse impacts of CO_2_ and other pollutant emissions from coal combustion to the environment have become a major public concern globally. Developing advanced technologies for production of affordable and low emission coal-derived power is thus imperative to a sustainable economy both domestically and globally [1]. Some of the developing technologies for future coal-firing power plants include the high-efficiency ultra-supercritical steam cycle with reduced of CO_2_ emissions, oxy-firing for economical post-combustion CO_2_-capture, and the integrated gasification combined cycle with pre-combustion CO_2_-capture [2,3,4]. In these emerging technologies, real-time monitoring of the process conditions and equipment physical states are highly desired for realizing intelligent operation to enhance the energy efficiency and safety assurance of the power plants. Temperature is one of the most important parameters needed for process optimization and control as well as for equipment health monitoring and operational management. Also, for industrial scale reactor design and process control, it is often necessary to have the information on temperature distribution over large-size equipment and transportation lines. Unfortunately, current sensors such as the conventional thermocouples are inefficient for direct deployment and operation in many critical locations in coal-firing power plants, e.g., gasifiers and combustors, involving high-temperature, high-pressure, and highly erosive and corrosive environments. 

In the past few decades, fiber optic sensors (FOS) have been under development and shown potential for high temperature applications due to their unique advantages such as compactness, high resolution, remote operability, multiplexing capability, and most importantly, good survivability in harsh environments because of their ceramic nature [5,6,7,8,9,10,11]. The optical fiber Bragg gratings (FBGs) have been particularly investigated for high resolution, multiplexed sensing applications since their first demonstration in 1978 [12,13,14]. However, FOS have encountered major challenges in achieving adequate sensor protection, packaging, installation, and thermal stability because of their inherent material brittleness, very small sizes, and inevitable high-temperature solid-state diffusion across functional optical interfaces. 

**Figure 1:**
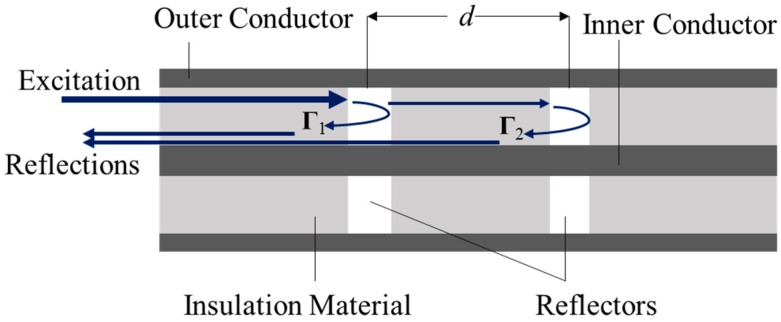
Diagram of CC-FPI sensor with a single pair of reflectors.

More recently, Xiao and coworkers [15,16,17] demonstrated a new coaxial cable Fabry-Pérot interferometric (CC-FPI) sensor of which the operating mechanism is quite similar to that of the well-established optical fiber interferometric sensor. Because of their relatively large size, flexibility, and the mechanical strength of the constituting materials, coaxial cables can tolerate much larger strain and forced deformation and resist mechanical or vibrational impacts even without additional packaging or protection. The principle of the CC-FPI sensor operation is schematically illustrated in Figure 1, where two identical weak reflectors are inserted into the insulator along the cable. The two waves Γ_1_ and Γ_2,_ as seen in Figure 1, are reflected from the reflectors interfere to generate signal (*U*), which can be expressed by the following equation [16]

(1) $$U=2\cdot \text{Γ}(f)e^{-\alpha z}\cos\left(2\text{π}f\frac{2d\sqrt{{\text{ε}}_{r}}}{c}\right)\cos\left[2\text{π}f\left(t+\frac{2d\sqrt{{\text{ε}}_{r}}}{c}\right)\right]$$

where Γ(*f*) is the amplitude coefficient of the reflection; *f* is microwave frequency; *α* is the propagation loss coefficient; *z* signifies the cable direction; *t* is the time; *d* is the distance between two reflectors; *ε_r_* is the relative permittivity of the insulation material; and *c* is the speed of light in vacuum. 

As described by Equation (1), the resultant interferogram depends on the inter-reflector distance (*d*) and relative permittivity (*ε_r_*) of the insulator. The previous work of Xiao and coworkers used commercial MW communication coaxial cables on which multiple pairs of weak reflectors were created by drilling periodical holes through the outer conductor and dielectric insulation layer along the cable [15,16]. These simple multi-point CC-FPI sensors were successfully demonstrated for measurements of strain and low temperature variations through monitoring the interferometric signal (*U*), which changes by strain- or temperature-induced variations of *d* and/or *ε_r_* [15,16]. However, CC-FPI sensors that can be suitable for high temperature applications have not been explored to date because of the lack of specialty coaxial cables for operating in high temperature harsh environments. The present work focuses on the first development of a new type of metal-ceramic coaxial cable FPI (MCCC-FPI) sensor and demonstration of its capability for monitoring high temperature. 

## 2. Experimental Section

### 2.1. Materials

The following materials were used for constructing the MCCC-FPI sensors. Stainless steel tubes with 5.4 mm O.D. and 3.1 mm I.D. (Grade 316, ASTM A213/A269, McMaster Carr: Elmhurst, IL, USA) were used as outer metal conductor. Stainless steel wires with diameter of 1.34 mm (Grade 316L, ASTM A555/A580, McMaster Carr: Elmhurst, IL, USA) were used as inner conductor. The stainless steel tubes and wires were used as received after simply cleaning their surfaces by wiping with isopropanol. Dense open-ended extruded alumina tubes (3.05 mm O.D. and 1.35 mm I.D., 99.5% α-Al_2_O_3_, 95% density, Ceramic Solutions Inc., Conroe, TX, USA) were used as the dielectric insulator for the MCCC. The alumina adhesive (Ceramabond 503, α-alumina, AREMCO Inc., Valley Cottage, NY, USA) was used to fix the alumina tube insulator onto the inner conductor in some cases. The outer surface of the alumina tubes were polished by a diamond tipped 320 grit grinding wheel (UNIPOL—820, MTI Corp., Richmond, CA, USA) to finely adjust its diameter for fitting into the stainless steel tube. The basic physical properties of the materials used for the MCCC-FPI are given in Table 1.

**Table 1:** Important physical properties of the materials for MCCC.

	Stainless Steel (Grade 316)	α-Alumina	Air
TEC, 10^−6^ (^o^C^−1^)	8.9–11.1	8.4	(Ideal Gas behavior)
Relative permittivity, *ε_r_*	N/A	9.5~12 [18]	~1.0
Conductivity,	11.6 µΩ/cm	N/A	N/A
Maximum Operate temperature in air (^o^C)	700 [19]	1750	N/A

### 2.2. Sensor Fabrication 

Two FPI sensors with distinct reflector-insulator structures were fabricated. The first one was made by stringing three separate segments of alumina tube with the stainless steel wire to form two 2-mm-wide annular air gap rings in the insulation, which served as reflectors to form a MW interferometer. The three tubes were attached to the wire using alumina adhesive at a single point of each tube with attachment length of ~5 mm. Such a single-point attachment between each alumina tube and the SS wire prevents the alumina tubes from sliding away from their intended positions on the wire upon expansion and contraction during thermal cycles and meanwhile avoids development of severe stress to cause damages by mismatch of thermal expansion that may occur if the alumina tube is strongly attached to the wire over a large length. This alumina adhesive fixed tube-wire assembly was cured at room temperature for 4 h and subsequently hardened at 250 °C for 8 h before being mounted into the metal tube. Because the TEC of the stainless steel is larger than that of the alumina, the temperature-induced change of the inter-reflector distance *d* in this structure depends on the expansion or contraction of the metal wire. 

The second one was made by cutting two 2-mm-wide slots into the alumina tube to the depth of half the outer diameter and then inserting the metal wire. These half-circle slots acted as the reflectors and no physical attachment was created between the alumina tube and stainless steel wire. The entire alumina tube insulator with the half-circle slots is in single piece and its expansion or contraction is not affected by the metal wire. Thus, the temperature-induced change of *d* in this second structure is determined by the expansion or contraction of the alumina tube section in between the two slots. These alumina tube-metal wire assemblies were then inserted into the stainless steel tubes to form MCCC-FPI sensors. 

**Figure 2:**
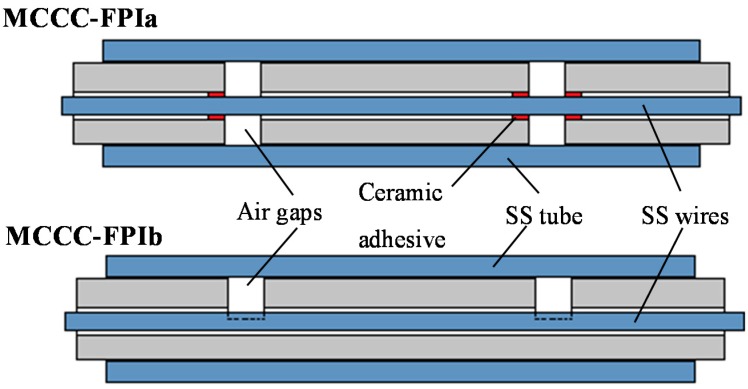
Schematics showing the structures of MCCC-FPIa and MCCC-FPIb sensors.

The first sensor is denoted as MCCC-FPIa and the second sensor is denoted as MCCC-FPIb hereafter. The structures of the two MCCC-FPI sensors are schematically illustrated in Figure 2. After the MCCC-FPI sensors were completely assembled, they were annealed in air at 700 °C for 48 h before being tested for high temperature measurement at 200–500 °C. The purpose of this pretreatment at a temperature much higher than the intended temperature application was to passivate the metal conductor surface by oxidation, relax mechanical stress introduced by tight mounting, and stabilize the microstructures of the metal and ceramic materials.

### 2.3. High Temperature Measurement 

The MCCC-FPI sensor measures temperature by monitoring the interferogram shift from a baseline, which can be defined at an arbitrary temperature (*T_0_*) of convenience. For a specific sensor, the interference signal (*U*) given by Equation (1) depends only on the product of distance *d* and the square root of the relative permittivity *ε_r_*, namely $2d\sqrt{{\text{ε}}_{r}}$. The distance *d* at temperature *T* (*d_T_*) is commonly expressed by

*d_T_ = d_0_ + d_0_ ×* β*_T_ ×* (*T−T_0_*) (2)

where *β_T_* is the TEC of the stainless steel wire for MCCC-FPIa or the alumina tube for MCCC-FPIb. The temperature-dependent *ε_r_* is often given as a polynomial function of *T* [20]:

(3) $${\text{ε}}_{r,T}=\sum_{i=0}^{n}\left(a_{i}\times T^{i}\right)$$

where *a_i_* (and *a_0_* = *ε*_r_(T_0_)) are the constant coefficients. The frequency shift (Δƒ) of the reflection signal given by Equation (1) then only responds on temperature when no chemical or structural changes are involved in the cable materials. Thus, the temperature change can be measured by monitoring the Δƒ.

**Figure 3:**
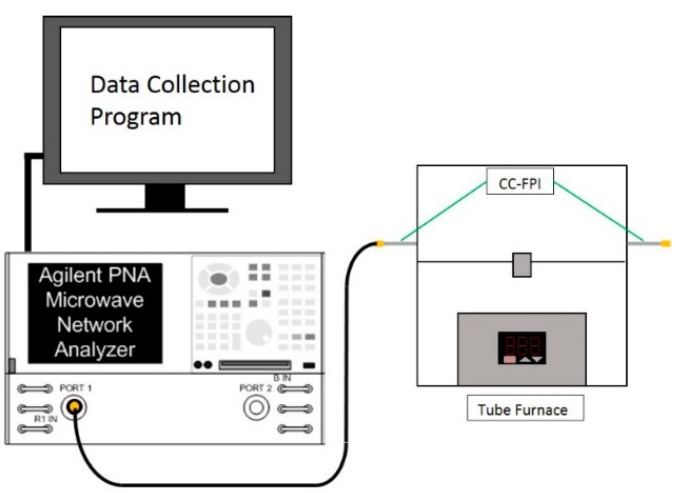
Schematic diagram of the apparatus for high temperature MCCC-FPI sensor test.

After the high temperature pretreatment, the MCCC-FPI sensors were examined for temperature monitoring using an apparatus as schematically shown in Figure 3, which is similar to the experimental system described in previous publications [15,16]. The MCCC was hosted in a temperature-programmable tubular furnace (Lindberg Blue M) with the FPI sensing section located at the center of the furnace where the temperature control stability is ±1.0 °C. A high accuracy thermocouple (Type K TP 741, HD 2178.2, accuracy ±0.004·*t*, *t* is temperature of measurement junction in °C, and measuring range −100–800 °C, Delta Ohm) was installed at the location of the FPI sensor for actual temperature reading. A vector network analyzer (Agilent 8363-B VNA) was used to continuously scan and record the MW interferogram reflected from the FPI when temperature was varied. The VNA was configured with an observation bandwidth from 2 GHz to 6 (or 8) GHz with 12,001 sampling points (minimum step is 1 Hz if needed) and scanning the spectrum takes less than a second. After Fourier transfer function on the VNA was applied, the signal was gated in time domain in order to focus on the two reflections (air gaps) of the cable and suppress external reflections such as those generated at the terminal ends and the connections between the MCCC and data communication coaxial cables. A computer workstation was connected to the VNA for data acquisition and processing. The MCCC-to-(communication cable) connection was located outside of the furnace that is both convenient for sensor installation and desirable for mitigating mechanical stress development under thermal expansion mismatches at the joints of connectors, SS conductors, and alumina tube insulators.

## 3. Results and Discussion

### 3.1. Sensor Structures and Response to Temperature 

**Figure 4:**
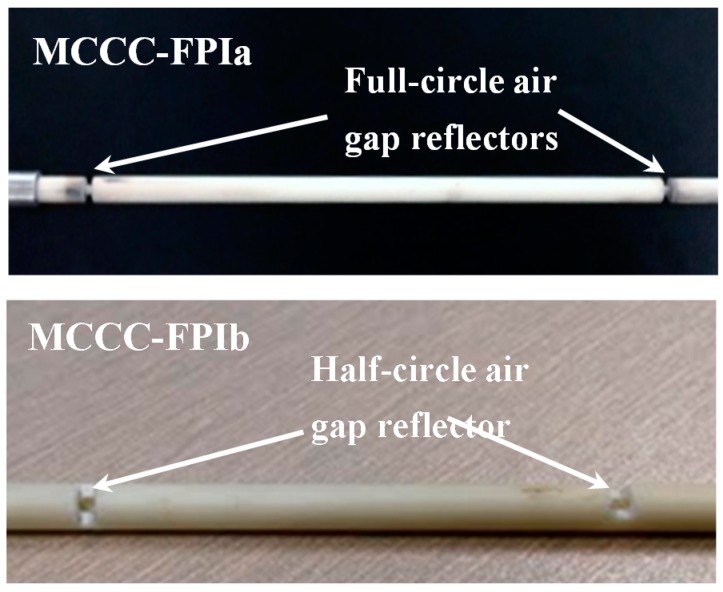
Photographs showing the air gap reflectors formed by alumina tube insulators in the MCCC-FPI sensors.

Figure 4 shows the photographs of the two types of air-gap reflectors formed by the alumina tube insulators in the MCCC-FPIa and MCCC-FPIb sensors. The inter-reflector distances (*d*) for MCCC-FPIa and MCCC-FPIb were 10 cm and 7.5 cm, respectively; and the air gap width was 2 mm for both sensors.

**Figure 5:**
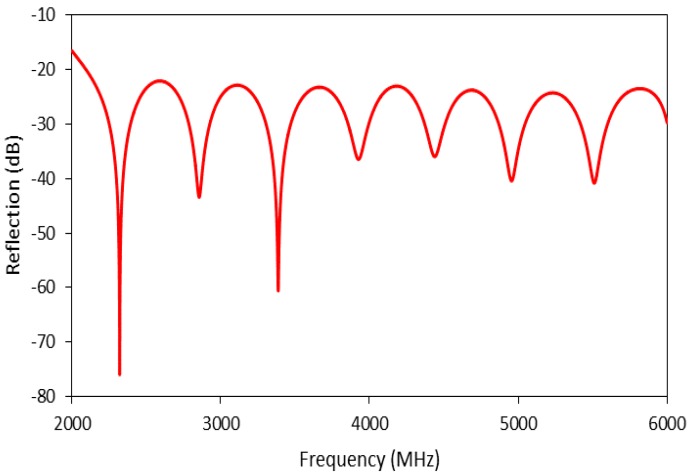
The reflected interferogram of MCCC-FPIa.

The sensors were first scanned for reflection spectra from 2 GHz to 8 GHz to examine their effectiveness as MW interferometers. Both MCCC-FPI sensors generated sharp reflection peaks with excellent signal-to-noise ratios that are appropriate for accurate measurement of Δƒ upon temperature changes. The reflected MW spectrum for MCCC-FPIa is shown in Figure 5 as an example. In this study, the sharp peak at around 3.4 GHz in the baseline spectra was employed for monitoring the temperature-induced Δƒ for MCCC-FPIa. For the MCCC-FPIb sensor (spectrum not shown), the peak at about 7.1 GHz was used for Δƒ monitoring.

**Figure 6:**
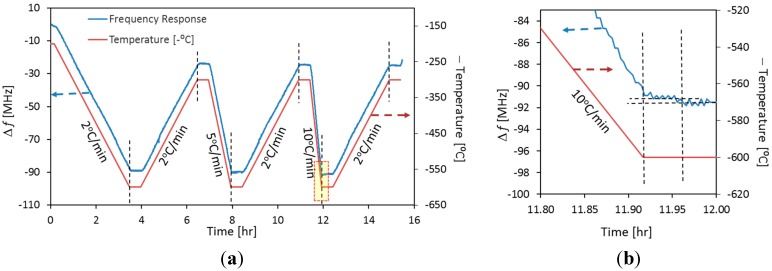
The frequency shift (Δƒ) for MCCC-FPIa as a function of time in response to the programmed temperature change (temperature switched sign for better visual comparison): (**a**) the entire temperature program; (**b**) an enlarged section showing transition from 10 °C/min heating to dwelling at 600 °C.

Experiments were also conducted to estimate the sensors’ response time to environmental temperature changes. Figure 6 shows the Δƒ for MCCC-FPIa as a function of time in response to the programmed temperature change of the furnace. The temperature ramping rate was varied at 2, 5, and 10 °C/min while the cooling rate was kept 2 °C/min in all cases to ensure that the naturally cooled chamber temperature could match up with the programmed cooling rate. The spectrum was scanned five times per minute. Results in Figure 6 indicate that the MCCC-FPI responded very fast to temperature changes and the response time was less than 180 s even for a heating rate of 10 °C/min according to the comparison between the time for ramping-to-dwelling transition in the temperature program and the time of the sensor’s frequency stabilization at the corresponding temperature transition point. This short response time may be attributed to the fast and highly efficient heat transfer by conduction through the metal and dense ceramic materials as they are in physical contact in the sensor structure. Such a fast sensor response is desirable for near-real-time *in situ* monitoring.

### 3.2. High Temperature Measurement 

**Figure 7:**
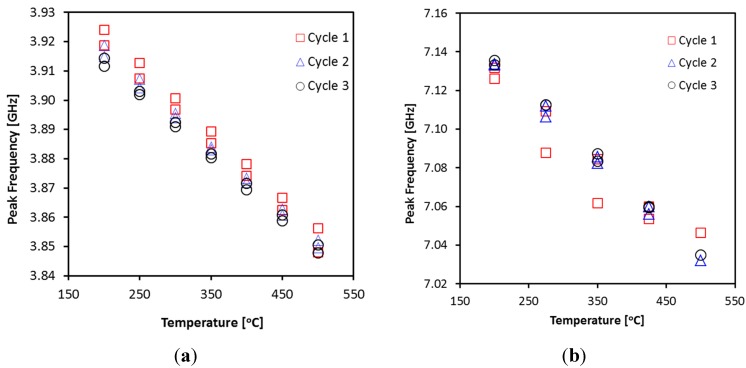
Evolution of the relationship between peak frequency and temperature during the pretreatment of first three heating-cooling cycles for MCCC-FPIa (**a**); and MCCC-FPIb (**b**).

The temperature measurement test was performed for both sensors in a temperature range of 200–500 °C. Prior to the temperature sensing tests, the sensors were subjected to five thermal cycles after they were connected to the instrument and mounted in the furnace. In each thermal cycle, the temperature was first ramped up to 600 °C, followed by a 30-min dwelling step, and then cooled down to 200 °C at heating and cooling rates of 5 °C/min. Such a multi-cycle heat treatment was found necessary for structural stabilization of the sensor components and for relaxation of mechanical stress introduced in the assembling process that could cause measurement inconsistency if not pretreated. Figure 7 shows the stabilizing relationships between peak frequency and temperature during the first three thermal cycles for both MCCC-FPIa and MCCC-FPIb. Further improvement in signal (frequency) consistency during heating and cooling became rather small in the fourth and fifth cycles and are thus not included in the figure for clarity. In all experiments of sensing test, the heating and cooling rates were also 5 °C/min and the reflection spectrum was taken after 10–30 min dwelling at each designated temperature. 

Figure 7 compares the experimentally measured Δƒ as a function of temperature for the MCCC-FPIa and MCCC-FPIb sensors. Data shown in Figure 7 include the values measured both ways of heating and cooling processes. An overnight dwelling at ~500 °C was used before the measurement of cooling down process so that the measurement in heating and cooling processes can be considered independent of the measurement taken during the heating-up process. For simple evaluation of the measurement deviations, only the experimental data measured during the heating-up process were used to correlate the temperature-dependences of the Δƒ for both sensors; and the data obtained during cooling process were employed for estimating the measurement deviations. 

**Figure 8:**
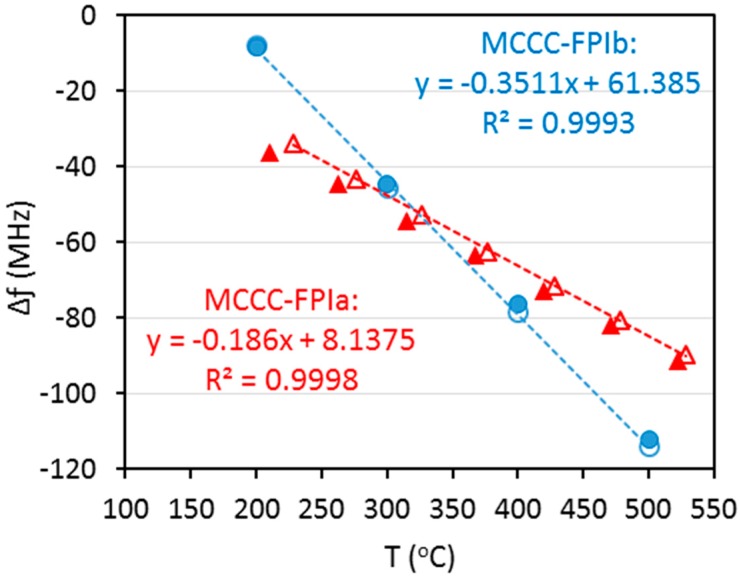
Experimentally measured frequency shift Δƒ as a function of temperature for MCCC-FPIa and MCCC-FPIb with linear correlations shown by dashed lines: ∆ and ▲ represent MCCC-FPIa; ○ and ● represent MCCC-FPIb; ∆ and ○ represent data of heating process used for correlation; ▲ and ● represent data of cooling process.

Based on the correlations between Δƒ and temperature shown in Figure 8, the temperature-dependences of *Δƒ*, in terms of “per degree frequency shift”, was found to be −0.186 MHz/°C for MCCC-FPIa and −0.351 MHz/°C for MCCC-FPIb. This means that the sensitivity of MCCC-FPIb sensor is higher than that of the MCCC-FPIa sensor. This is likely due primarily to the higher frequency of the peak employed by the MCCC-FPIb. According to Equation (1), the interferogram phase shift depends on the value of (*f × d_T_ × ε_r,T_^0.5^*). If resonant peaks of same frequency are used for both sensors, the MCCC-FPIa is expected to have higher sensitivity because its d_0_ and β_T_ of stainless steel wire, which determines the d in MCCC-FPIa, are greater than those of the alumina tube, which determines d in MCCC-FPIb. Since the same insulator material was used in both sensors, *i.e*., identical *ε_r,T_*, the larger *d_0_* and *β_T_* would cause greater changes of (*f × d_T_ × ε_r,T_^0.5^*) value, leading to a larger phase shift for MCCC-FPIa than for MCCC-FPIb in response to the same temperature change. However, the peak frequency used for MCCC-FPIb is ~2.1 times that used for MCCC-FPIa while the difference of changes in (*d_T_ × ε_r,T_^0.5^*) between the two sensors is comparatively smaller because of the very small β_T_ values and weak temperature dependences of *ε_r,T_* in the high frequency ranges [18,21]. Thus, comparison of temperature sensitivity between the current two sensors is dominated by the difference in peak frequency used rather than the material and structural parameters.

Since the VNA has a frequency scanning resolution (1 Hz) much smaller than 0.1 MHz, both sensors are in principle capable of monitoring temperature changes within 1 °C. When comparing the Δƒ data measured during cooling process to the values calculated by the Δƒ~T correlations obtained from the data of the heating process, the average absolute deviations were 7.7% and 2.7% for MCCC-FPIa and MCCC-FPIb, respectively. Moreover, the distributions of measurement deviation for the two sensors are fundamentally different. The MCCC-FPIb had a normal distribution of small positive and negative deviations; but the MCCC-FPIa sensor exhibited systematically negative deviations. The systematic deviation of MCCC-FPIa was likely caused by position shift of the individual alumina tube segments, which were found to loose from the SS wire after a few heating-cooling cycles which caused breakup of the single-point ceramic adhesion due to TEC mismatch between the (alumina tube)/(alumina adhesive)/(SS wire). 

The above results of temperature measurements indicate that the MCCC-FPIb sensor outperformed the MCCC-FPIa with smaller deviation and much better measurement consistency because the former had a solid structure of one-piece ceramic combining the reflectors and insulator while no strong adhesion existed between the tube and wire. Thus, position changes between the insulators and air gap reflectors are avoided and TEC mismatch-caused thermal stress between the insulator and metal conductors is essentially nonexistent in the MCCC-FPI sensor. Thermal drifting of sensing signal was also not appreciable for the MCCC-FPI sensors throughout the operation at 200–500 °C for more than one week. However, the thermal stability test for longer periods and examination of the sensor behavior at temperatures above 500 °C are yet to be performed. 

## 4. Conclusions

A metal-ceramic coaxial cable Fabry-Pérot interferometer (MCCC-FPI) has been successfully fabricated using stainless steel materials for outer and inner conductors, dense α-alumina tubes as dielectric insulators, and two air gaps in the insulator as MW reflectors. The MCCC-FPI sensor has been demonstrated for measuring temperature between 200 and 500 °C using MW as sensing signal. The temperature measurement is achieved by monitoring the frequency shift (Δƒ) of a selected resonant peak of the MW interferogram reflected from the two weak reflectors. The MCCC-FPI sensor exhibited excellent linear temperature-dependence for Δƒ, good accuracy (deviation ±2.7%), and reasonable response speed. Thermal drifting of the sensing signal was also rather minimal for the MCCC-FPI sensors after operating for more than one week below 500 °C. Although further investigations on the thermal stability over longer times and sensor performance at above 500 °C are still ongoing, the MCCC has shown the potential for developing multipoint FPI sensors in a single-cable to achieve *in situ* and continuous monitoring of distributed temperature in harsh environments. 
